# Background of fatal pulmonary embolism: an analysis of all diagnosed fatal pulmonary embolism in 2015–2018 from Hospital District of Helsinki and Uusimaa

**DOI:** 10.1007/s11239-021-02550-z

**Published:** 2021-08-16

**Authors:** Sane Markus Antero

**Affiliations:** Heart and Lung Center, Helsinki University and Helsinki University Hospital, Stenbäckinkatu 9, Lumivaarantie 23A, 02140 Espoo, Helsinki, Finland

**Keywords:** Pulmonary embolism, Cardiac arrest, Prevention, Mortality

## Abstract

Pulmonary embolism (PE) mortality has decreased in the last decades but acute PE is still associated with significant fatality. Specific information on fatal PE patients could guide how to efficiently improve PE management but to date this information has been scarce. All the individuals with PE defined as an immediate or underlying cause of death were collected from the death certificate archive of the Hospital District of Helsinki and Uusimaa, Finland, including approximately 1.7 million inhabitants (2015–2018). Crude and age-adjusted mortality rates and proportional mortality were calculated, and the distribution of comorbidities at death and death location (in-hospital, palliative care, or out-of-hospital) was analyzed. In total, 451 individuals with fatal PE were identified (238 females) with a mean age of 72 years (SD 13.5 year). Most of the fatal PEs (n = 264, 54.5%) occurred out-of-hospital and surprisingly, 70 (26.5%) of these individuals s had a history of mental illness or substance abuse. The out-of-hospital resuscitation was attempted in 108 (40.1%) individuals but only 7 (6.5%) received thrombolysis during resuscitation. Fatal PE occurred during hospitalization in 98 individuals and in 54 (55.1%), the diagnosis was only made postmortem. Majority of the fatal PEs occurred out-of-hospital and were diagnosed postmortem whereas only small proportion of deaths occurred to in-hospital PE patients. The earlier diagnosis of PE, which may be accomplished by raising the general awareness of PE, is necessary to prevent these sudden deaths of whom many occurred to individuals with history of mental illnesses or substance abuse.

**Supplementary Information:**

The online version contains supplementary material available at 10.1007/s11239-021-02550-z.

## Highlights

- Most fatal pulmonary embolism occur suddenly out-of-hospital
- Most fatal pulmonary embolism are diagnosed only postmortem
- Thrombolysis is rarely used in out-of hospital cardiac arrests caused by pulmonary embolism
- The prevention of fatal pulmonary embolism is difficult without improving the early detection which may be accomplished by increasing the general awareness of pulmonary embolism

## Introduction

Pulmonary embolism is the third most common cause of cardiovascular mortality and its incidence has increased along with the aging of the population in the developed countries [1, 2]. Fortunately, studies have reported a decrease in PE mortality in the last decades both in the European region and in North America [3, 4]. However, an alarming rebound increase was observed among young and middle-aged adults in the USA after 2006. Further advances in the management of PE are needed to continue the positive trend and better understanding of PE fatalities is warranted.

Multiple methods for thromboprophylaxis for the prevention of PE exist [5] and the hospital care after the diagnosis is optimized by recognizing the high-risk PE patients with prediction tools [6, 7]. On the other hand, PE is likely the underlying cause in 5% of the out-of-hospital cardiac arrests and often the fatal PEs are only diagnosed postmortem [8, 9]. Unfortunately the overview of all fatal PE patients has not been previously presented and therefore it remains unclear that what proportion of patients would have benefitted from adequate thromboprophylaxis or improvements made in PE -diagnostics or -hospital care.

The aim of this study was to analyze all diagnosed deaths from PE for the period 2015–2018 in the largest hospital district in Finland and describe the distribution of place of death and patient characteristics to identify new epidemiological trends and information potentially useful to improve prevention of fatal PE.

## Methods

### Study population

The HUS district has a population of approximately 1.7 million in 2018, (mean age 40.8 year) [10, 11]. The healthcare system in Finland is universal with excellent coverage and high performance in the WHO surveys [12]. The quality of civil registration and vital statistics systems (including death certificate registry) has been also shown to be very high in Finland [13].

### Data source

All the individuals from the HUS district with fatal PE event (individuals with 10th revision of International Classification of Diseases (ICD code) I26.0 or I26.9 for PE as their immediate or underlying cause of death) were identified from death certificate archive, which is produced by the Statistics Finland [14]. The information of all deaths of persons permanently resident in Finland at the time of their death is stored in the death certificate archive. The production of statistics and archiving of death certificates is based on the national Act (1973/459) and Decree (1973/948) on the investigation of the cause of death, and European Commission Regulation No 328/2011 concerning the statistics on causes of death that EU Member States have to deliver.

Filling out the death certificate is mandatory for every deceased person. The death certificate includes vital information such as the social security number, the place of residence, time of death and the diagnosis for underlying and immediate cause of death. Also a summary of the medical history of the deceased and the events leading to death are included. In Finland, autopsy is mandatory in a case of sudden death or death preceded by surgery, among other cases. In comparison the autopsy rate in Finland is higher than in most other EU member states which data is available from World Health Organization report and on average in 2015 the autopsy rate was 13.2% in the Member states of European Union and 21.1% in Finland. [15, 16].

In Finland, it is common that PE is often defined as immediate cause of death if any other illnesses or factors exist that can cause or increase the risk for PE and only idiopathic PEs are defined as underlying cause of death. For example if surgery or trauma precedes PE or previous cancer diagnosis exists these are determined as underlying cause of death and PE as immediate. In addition co-existing disease that increase the risk of fatal outcome of PE such as coronary artery disease can be defined as underlying cause of death and PE as immediate one.

The death certificates of the individuals were available as electronic documents. The detailed information of each identified individuals with ante or postmortem diagnosed fatal PE was collected from the electronic patient record system. For individuals for whom death occurred outside HUS district, the details of the events leading to death was acquired from the brief summary in death certificate and from the common patient record system in Finland that combines all the information from different electronic patient records systems nationwide [17].

The following information was collected: the place of death (out-of- or in-hospital), the method for PE diagnosis, was PE diagnosis made ante or postmortem, was anticoagulation used before PE death, was resuscitation attempted and was thrombolysis used during resuscitation, sex and age of the subject, previous surgery or inpatient hospital care in the preceding 3 months, the information on previous VTE including the manifestation, etiology and time since previous diagnosis. The risk factors for previous PE were categorized as strong, moderate or weak as presented in the latest guideline by European society of cardiology [18]. The co-morbidities were also evaluated and the information on the previously diagnosed high blood pressure, cancer, respiratory illness (Asthma or Chronic obstructive pulmonary disease [COPD]), coronary artery disease, mental illness (established diagnosis of depression, schizophrenia or/and personality disorders in the electronic patient records) and substance abuse. The information of underlying causes of death in individuals with PE as immediate cause of death was also collected.

The yearly incidence data of PE was collected from the hospital discharge data of Care Register for Health Care which is database controlled by National institute for Health and Welfare [19]. This database collects social security code linked information of both inpatient and outpatient use of health services and all the patients with PE ICD code I26.0 or I26.9 as their main discharge diagnose were collected from 2015 to 2018.

### Definitions for the subgroup analyzed

The cohort was divided as followed on the base of the place of death: (1) Out-of-hospital, (2) In-hospital and (3) In palliative care.

The intensity of the treatment was evaluated from the patient’s records and if patient’s records clearly stated that patient was admitted to end stage care either in palliative ward or home the death was considered to occur in palliative care.

#### Ethics

The study permission was approved by study committee of Heart and Lung center of Helsinki University Hospital and ethics committee decided that ethical permission is not needed.

### Statistics

All statistical analyses were performed with IBM SPSS 25.0 for Windows (IBM Corp., Armonk, N.Y., USA). Data are presented as means (with standard deviation for normally distributed continuous variables) and proportions (%). However the time from PE diagnosis and previous VTE to death were reported in median as the data were skewed and interquartile range is also presented. The normality of continuous variables was assessed by the Kolmogorov–Smirnov test. The independent sample T-test was used to evaluate the difference between individuals with mental illness or substance abuse to other individuals who died out-of-hospital. A p value of < 0.05 was considered statistically significant.

## Results

In total 451 individuals with fatal PE event were identified during 2015–2020. Four patients (0.8%) were located outside HUS district when fatal PE event occurred. Characteristics of the population are summarized in Table 1 and 5-year age distribution in supplementary appendix Table 3.

**Table 1:** Mortality data, the annual number and the characteristic of the individuals with fatal PE event from HUS district (2015–2018)

	Total	2015	2016	2017	2018
Patients with fatal PE event, n	451	110	114	121	106
Population, n (All deaths, n) in HUS district^a^		1,620,261 (11,835)	1,638,293 (12,070)	1,655,624 (12,042)	1,671,024 (12,225)
Unadjusted mortality per 100,000 people		6.8	7.0	7.3	6.3
Age standardized mortality per 100,000 people^b^		8.4	8.2	8.4	7.4
PE or DVT defined as an underlying cause of death		38	38	56	41
PE defined as immediate cause of death		72	76	65	65
Proportionate mortality		0.93%	0.94%	1.0%	0.87%
Male/Female, n (%)	213/238 (47.2/52.8)	52/58 (47.3/52.7)	50/64 (43.9/56.1)	65/56 (53.7/46.3)	46/60 (43.4/56.6)
Age, y, mean (± SD)	72.3 (13.5)	73.2 (12.9)	73.3 (12.9)	69.5 (14.7)	73.3 (13.6)
BMI, kg/m^2^ mean (± SD)	29.7 (9.1)	29.9 (9.2)	30.3 (11.0)	30.6 (9.3)	28.3 (7.0)
PE diagnosed in					
Autopsy, n (%)	344 (76.3)	84 (76.3)	90 (78.9)	88 (72.7)	82 (77.4)
Imaging, n (%)	69 (15.3)	17 (15.5)	14 (12.3)	22 (18.2)	16 (15.1)
Clinically, n (%)	38 (8.4)	9 (8.2)	10 (8.8)	11 (9.1)	8 (7.5)
Cancer, n (%)	92 (20.4)	22 (20.0)	24 (21.1)	26 (21.5)	20 (18.9)
Coronary artery disease, n (%)	53 (11.8)	22 (20.0)	11 (9.6)	11 (9.1)	10 (9.4)
COPD/asthma, n (%)	72 (16.0)	21 (19.1)	13 (11.4)	19 (15.7)	19 (17.9)
Hypertension,n (%)	229 (50.8)	48 (44.5)	60 (52.6)	57 (47.1)	64 (60.4)
Mental illness, n (%)	45 (10.0)	9 (8.2)	8 (7.0)	17 (14.0)	11 (10.4)
Substance abuse, n (%)	53 (11.8)	9 (8.2)	13 (11.4)	13 (10.7)	18 (17.0)
Dementia, n (%)	77 (17.1)	21 (19.1)	20 (17.5)	15 (12.4)	21 (19.8)
Previous VTE, n (%)	62 (13.7)	14 (12.7)	16 (14.0)	22 (18.2)	10 (9.4)
Previous surgery or hospital treatment in past 3 months, n (%)	100 (22.2)	18 (16.4)	19 (16.7)	30 (24.8)	23 (21.7)
Smoking, n(%)	71 (15.7)	13 (11.8)	15 (13.2)	24 (19.8)	19 (17.9)

^a^Population in HUS district at the end of each year [14]

^b^EU 2013 standard population used [20]

Patients who died out-of-hospital were younger than patients who died in-hospital or in palliative care (68.5 year vs. 75.1 year vs. 80.0 y respectively, p < 0.001).

In total, 100 patients (22.2%) had a previous history of surgery or hospitalization on average 28 or 23 days beforehand, respectively. The appropriate prophylaxis was used in 43 (43%) of these patients. Overall, 62 (13.7%) of the patients had a history of VTE (32 PE, 24 DVT and 6 thrombophlebitis) and 37 (58.7%) of these VTEs were classified as caused by no or only weak risk factor. The median time since the previous VTE was 3 years (IQR 1.5–9 years). The most common underlying causes of death in individuals with PE as immediate cause of death were heart disease including coronary artery disease and heart failure (n = 98, 27%), deep venous thrombosis (n = 90, 25%), cancer (n = 80, 18%), dementia (n = 32, 7%) and external causes (n = 26, 6%).

The characteristics of the patients died out-of- or in-hospital or in palliative care presented in Table 2

**Table 2:** Characteristics of individuals with fatal PE event divided on the basis of the place of death

	Died out-of-hospital	Died in-hospital	Died in palliative care
Total number, n (%)	264 (58.5)	98 (21.7)	89 (19.8)
PE or DVT as underlying/immediate cause of death	126/138	34/64	16/73
Male/Female, n (%)	135/129 (51.1/48.9)	41/57 (41.8/58.2)	37/52 (41.6/58.4)
Age, y, mean (± SD)	68.5 (13.7)	75.1 (12.1)	80.0 (10.6)
BMI, kg/m^2^ mean(± SD)	30.1 (9.4)	30.8 (10.5)	27.4 (5.4)
PE diagnosed in (%)			
Autopsy, n (%)	252 (95.5)	54 (55.1)	38 (42.7)
Imaging, n (%)	4* (1.5)	27 (27.6)	38 (42.7)
Clinically, n (%)	8 (3.0)	17 (17.3)	13 (14.6)
Cancer, n (%)	31 (11.7)	19 (19.4)	42 (47.2)
Coronary artery disease, n (%)	21 (7.9)	16 (16.3)	16 (18.0)
COPD/asthma, n (%)	38 (14.4)	18 (18.3)	16 (18.0)
Hypertension, n (%)	119 (45.0)	67 (68.4)	43 (48.3)
Mental illness, n (%)	30 (11.3)	12 (12.2)	3 (3.3)
Substance abuse, n (%)	40 (15.1)	8 (8.1)	5 (5.6)
Dementia, n (%)	33 (12.5)	18 (18.4)	26 (29.2)
Previous VTE, n (%)	35 (13.2)	18 (19.4)	9 (10.1)
Previous surgery or hospital treatment in the preceding three months, n (%)	36 (13.6)	34 (34.7)	20 (22.4)
Smoking, n (%)	46 (17.4)	12(12.2)	13 (14.6)
Resuscitation attempt, n (%)	108 (41.2)	50 (51.0)	2 (2.2)
Thrombolysis during resuscitation, n (%)	7 (6.4)	14 (28.0)	0 (0)

*Previously established PE diagnosis with TT-angiography (n = 3) or Echocardiography during resuscitation (n = 1)

### Out-of-hospital fatal PE events

Majority of the patients (58.3%, n = 264) died out-of-hospital and the diagnosis of PE was mainly made postmortem (95.4%, n = 252) (Table 2). Of these patients (58.7%, n = 155) were found dead and of the remaining 109 patients, a resuscitation was attempted in 108 patients (99%) by paramedics. In seven patients (6.4%) the PE was clinically suspected and systemic thrombolysis was used during resuscitation. Thirty five patients (13.2%) had a history of previous VTE and 24 (68.5%) of these were considered to be caused by no or only weak risk factors. Three patients were diagnosed with PE and one with DVT on average 29 days before the death and these patients died despite appropriate anticoagulation was prescribed.

Sixty nine patients (26.1%) had a history of mental illness or substance abuse (mainly alcohol). These patients were younger than other patients who died out-of-hospital (61.8 y vs. 71.0 y,p < 0.001) and the majority of these patients were found dead (74.3%, n = 52).

### In-hospital fatal PE events

Ninety eight patients (21.7%) were in-hospital while death occurred and the type of hospital was University Hospital in 19 (19.4%), Central Hospital in 25 (25.5%), Regional hospital in 35 (35.7%) and Health center hospital ward in 19 (19.4%) of the cases. PE diagnosis was made antemortem in 27 (27.5%) of these patients and fatal PE event occurred despite appropriate hospital care with therapeutic anticoagulation.

In addition, the PE was clinically suspected in 17 patients and eight of these were also anticoagulated. The median time from the PE diagnosis to death was 2 days (IQR 1–3 days)). In 54 patients (55.1%) the PE diagnosis was made only postmortem. The indications for hospital care were diverse but in 11 (18.5%) patients a working diagnosis was infection and also 12 (22.2%) patients were in hospital during post-surgical rehabilitation. Nine (16.7%) of these patients died in the emergency department before definite PE diagnosis was made. Of all the patients who died during hospitalization, the resuscitation was attempted in 50 (51.0%) of the patients and thrombolysis was used in 14 (28.0%) of these patients.

### Fatal PE events in palliative care

Eighty nine patients (19.7%) died in palliative care due to incurable permanent illness such as cancer (47.2%, n = 42) or dementia (29.2%, n = 26). The diagnosis of the PE was made premortem in 51 (57.3%) of the patients and anticoagulation was used in 40 (78.4%) of these patient. Postmortem diagnosis was made in 38 (42.7%) of these patients.

## Discussion

This study presents detailed data of all the diagnosed fatal PE events in four consecutive years that occurred in HUS district with population of approximately 1.7 million. In total, 451 deaths where PE was defined as immediate or underlying cause of death were analyzed. The results showed that majority of the fatal PE events (58.5%, n = 264) occurred out-of-hospital and overall majority of all fatal PE events were diagnosed only postmortem (76.2%, n = 344). This highlights the need for earlier diagnosis of PE and the recognition of PE as the etiology of out-of-hospital cardiac arrest whereas further advances in the care of hospitalized PE patients may have less impact on PE mortality.

Majority of the fatal PE events occurred out-of-hospital indicating that the further improvements in hospital care alone are insufficient to markedly decrease PE mortality. Interestingly as averagely 1500 PEs are diagnosed annually in HUS district the first symptom of PE is sudden death for approximately 5% of all PE cases. Few details of these patients are noteworthy. Firstly, the identification of PE from out-of- hospital cardiac arrest needs to be improved as in only minority (6.4%, n = 7) of the cases the PE was suspected and guideline recommended thrombolysis used during resuscitation [18]. As the previous data shows that the survival in the thrombolysis group was only 16% [21], it can be speculated that overall the use of thrombolysis in cardiac arrests caused by PE was minimal in the HUS district during this period. Secondly, certain characteristics of the patients were startling as more than quarter of the individuals had either diagnosed mental illness, such as severe depression, schizophrenia or history of substance abuse, whereas the prevalence of these illnesses is much lower in general population in Finland [22]. It is widely known that individuals with mental illnesses or substance abuse have lower life expectancy [23, 24] but the association of these diseases with the risk of fatal PE has not been previously described. These illnesses are not recognized as individual risk factors for PE [18] and specific pathophysiologic mechanism that promotes the development of fatal PE in these individuals is lacking. Possible explanation could be that substance abuse and mental illnesses causes delay to seek care and thus the risk for missed PE leading to fatal PE might be increased. Interestingly it was seen that most of these individuals (n = 52, 74.3%) were found dead without signs of attempts to contact healthcare. Further studies are needed to verify these findings but the awareness of PE especially in this vulnerable population should be increased as it may lead to earlier diagnosis of PE with better prognosis.

In this study minority (21.7%, n = 98) of fatal PE events occurred during hospitalization. Approximately in half of these patients the PE diagnosis was made ante-mortem and median duration from the PE diagnosis to death was 2 days for hospitalized patients, which is in line with the previous PEITHO study [25]. The prognosis of these patients might have been improved by using the pulmonary embolism response teams (PERT) [26] proposed by the latest guideline [18] and it has been shown that the hospital volume has an impact to the PE prognosis when the data from RIETE registry were analyzed [27]. The patients whose PE was not suspected ante-mortem probably would have benefitted from earlier diagnosis but unfortunately this study is unable to specify the causes for diagnostic delay. The wide variety of work-up diagnosis for these patients illustrates the challenges of PE diagnostics in-hospital patients. Although the proportion of missed fatal PE events of all fatal PE events in-hospital patients is considerable (55.1%, n = 54), this represents only a small minority of all in-hospital patients as in 2018 nearly 190,000 bed-days were registered in HUS district [28]. The low prevalence of missed fatal PE events and likely overall PE events of all in-hospital patients highlights the need for higher performing clinical decision rules used in combination with biomarkers to improve the recognition of PEs among in-hospital patients.

Finally, the observation that a fair proportion of fatal PEs (20%, n = 89) occurs to patients in palliative care is noteworthy and illustrates that PE can be considered to be a natural outcome of many incurable illnesses. This subgroup is most likely underestimated as it is coincidental to whom patients an autopsy was performed where many (42.7%, n = 38) of these fatal PEs were found. The future advances in PE management may not lead to overall decrease in PE mortality but if a shift from unexpected fatal PE events to fatal PE events in patients in palliative care is achieved, the advances can still be considered successive. On the other hand the efficacy of future advances in PE management can also be overestimated if the mortality reduction on population is only caused by the lack of exact cause of death definition for patients in palliative care.

### Strengths and weaknesses

This study described and analyzed a notable number of individuals with fatal PE event from a relatively large population cohort with universal health care and uniform electronic patient record systems which complemented the information from vital statistics. This data offers for the first time the overall picture of the fatal PE events and generates hypotheses for the most impactful measures to prevent PE deaths. As the characteristics of fatal PE cases were uniform in the four consecutive years it may be that the impact of specific interventions such as better recognition of PE as the etiology of out-of- hospital cardiac arrest may have permanent effect on the PE mortality. However, a number of limitations has to be considered.

Unlike previous epidemiological studies on PE mortality this study incorporated all the individuals with PE defined as immediate or underlying cause of death rather than individuals with PE or DVT as underlying cause of death [3, 4]. This method enables more accurate estimation of PE mortality [3, 4] and likely in addition with the higher autopsy rate in Finland explain why PE- and proportional mortality was higher in this data than in previous epidemiological studies [3, 4]. From a clinical perspective it is not relevant whether fatal PE was idiopathic or had any predisposing factor determined as underlying cause of death as the goal is to prevent all these cases and therefore it is reasonable to include all the fatal PEs when the overview of the background of these individuals is reported.

The data source may have limitations and certain selection bias in this data is likely present. In reality higher proportion of fatal PEs occur to old multi-morbid patients in palliative care to whom autopsy is not performed and thus the PE is ‘’missed’’ [8]. In terms of prevention, these fatal PE events are perhaps less important and focus should be on preventing especially sudden fatal PE events that occur out-of- or in-hospital for younger patients. Due to the practice patterns in Finland in the cause of death evaluation the sensitivity of identifying all fatal PE events especially in individuals under 65 years can be considered good. In Finland every death certificate is assessed for accuracy by a forensic pathologist and if the descriptions of the events leading to death and the defined causes of death are not considered to be causal, the death certificate is returned for corrections. This practice has been shown to improve the mortality statistics [29]. In addition, the autopsy rate in Finland is also higher than in many European Union counties where statistics are available [15]. The legislation in Finland defines that autopsy is mandatory if death has been sudden, unexpected or if surgical operation has preceded ensures that the definitive cause of death is determined to almost all individuals with sudden death out-of-hospital and with high-risk factors for PE. The proportion of autopsies performed of all deaths was 52% in individuals under 65 years in whole Finland in 2018 [16]. The fatal PE events reported in this study fulfill well the requirements of recent consensus statement of the definition of the PE death [30] as 76.3% of the PE deaths were category A1 (autopsy confirmed PE in the absence of another more likely cause of death), 15.3% category A2 (objectively confirmed PE before death in the absence of another more likely cause of death) and only 8.4% category A3 (PE is not objectively confirmed, but is most likely the main cause of death). Overall it is likely that the method used in this study for the collection of patients with fatal PE events ensures that representative cohort of these patients have been analyzed.

## Conclusion

This study showed that majority of fatal PE events occurred suddenly out-of-hospital. The unique observation of high prevalence of mental illnesses and substance abuse among fatal PE cases is also noteworthy and it seems that these patients are especially vulnerable to fatal PE. The PE can also be considered as a natural outcome of many incurable illnesses and thus in future the mortality statistics on population should report more specific data on PE deaths if the efficacy of future advances in PE management is evaluated.

## Supplementary Information

Below is the link to the electronic supplementary material.

Supplementary file1 (DOCX 12 kb)
