# Supplementary material for: Background of fatal pulmonary embolism: an analysis of all diagnosed fatal pulmonary embolism in 2015–2018 from Hospital District of Helsinki and Uusimaa
Source: J Thromb Thrombolysis. 2021 Aug 16;53(2):550–6. doi: 10.1007/s11239-021-02550-z (PMC8904330; doi:10.1007/s11239-021-02550-z)
Supplement: Supplementary file 1 — Supplementary file1 (DOCX 12 kb) [file 11239_2021_2550_MOESM1_ESM.docx]

|  | Year, the number of individuals with fatal PE  event in 5-year age group | | | |
| --- | --- | --- | --- | --- |
| Age (y) | 2015 | 2016 | 2017 | 2018 |
| 15-19 | 0 | 0 | 0 | 1 |
| 20-24 | 0 | 0 | 0 | 0 |
| 25-29 | 0 | 0 | 1 | 0 |
| 30-34 | 0 | 2 | 2 | 0 |
| 35-39 | 2 | 0 | 2 | 2 |
| 40-44 | 0 | 0 | 3 | 1 |
| 45-49 | 5 | 3 | 3 | 2 |
| 50-54 | 3 | 1 | 9 | 3 |
| 55-59 | 10 | 8 | 9 | 4 |
| 60-64 | 9 | 12 | 12 | 12 |
| 65-69 | 10 | 26 | 13 | 13 |
| 70-74 | 16 | 14 | 18 | 16 |
| 75-79 | 20 | 8 | 17 | 20 |
| 80-84 | 12 | 13 | 13 | 8 |
| 85-89 | 14 | 16 | 14 | 14 |
| 90-94 | 8 | 11 | 5 | 8 |
| 95-99 | 1 | 0 | 0 | 2 |

**Table 3. Individuals with fatal PE event in 5-year age group**
